# Role of developmental factors in hypothalamic function

**DOI:** 10.3389/fnana.2015.00047

**Published:** 2015-04-21

**Authors:** Jakob Biran, Maayan Tahor, Einav Wircer, Gil Levkowitz

**Affiliations:** Departments of Molecular Cell Biology, Weizmann Institute of ScienceRehovot, Israel

**Keywords:** homeostasis, neuroendocrine, Otp, SIM1, PAC1, SF-1, zebrafish model system, neuropeptides

## Abstract

The hypothalamus is a brain region which regulates homeostasis by mediating endocrine, autonomic and behavioral functions. It is comprised of several nuclei containing distinct neuronal populations producing neuropeptides and neurotransmitters that regulate fundamental body functions including temperature and metabolic rate, thirst and hunger, sexual behavior and reproduction, circadian rhythm, and emotional responses. The identity, number and connectivity of these neuronal populations are established during the organism’s development and are of crucial importance for normal hypothalamic function. Studies have suggested that developmental abnormalities in specific hypothalamic circuits can lead to obesity, sleep disorders, anxiety, depression and autism. At the molecular level, the development of the hypothalamus is regulated by transcription factors (TF), secreted growth factors, neuropeptides and their receptors. Recent studies in zebrafish and mouse have demonstrated that some of these molecules maintain their expression in the adult brain and subsequently play a role in the physiological functions that are regulated by hypothalamic neurons. Here, we summarize the involvement of some of the key developmental factors in hypothalamic development and function by focusing on the mouse and zebrafish genetic model organisms.

## Introduction

The hypothalamus is a key regulator of homeostasis in animals. It does so by integrating internal and external sensory signals, processing them, then exerting regulatory autonomic signals and neuroendocrine releasing peptides to maintain homeostasis (Pearson and Placzek, 2013). Hypothalamus-related neuropeptides were identified in ganglions of lower evolutionary animals such as corals and clams (Twan et al., 2006; Takayanagi and Onaka, 2010) and ontogenetic conservation of hypothalamus-related structures in the brains of Annelids and zebrafish has been demonstrated (Tessmar-Raible et al., 2007). In vertebrates, the hypothalamus resides ventrally to the thalamus, dorsally to the anterior pituitary and is structurally composed of several nuclei of interconnected cell populations. Each nucleus contains several neuronal types, and these work in an orchestrated manner within and between nuclei to regulate physiological functions including metabolism, water balance, satiety, reproductive physiology, circadian rhythm, and emotional responses (Machluf et al., 2011; Pearson and Placzek, 2013). Developmental abnormalities of the hypothalamus are associated with alterations in body growth and sexual development leading to adverse physiological and neurological conditions such as infertility, depression, chronic stress, autism and obesity (Michaud, 2001; Swaab, 2004; Silveira et al., 2010). Moreover, interaction of hypothalamic neurons with neighboring neuronal, astroglial and endothelial cells is highly important for sensing blood-borne hormones and metabolites. Failure to generate these interactions might lead to impairment in negative feedback signals, release of trophic neuropeptides and abnormalities in the structure of the neurohypophysial portal system (de Seranno et al., 2010; Gutnick et al., 2011).

Major efforts in recent years have been focused on the roles of hypothalamic transcription factors (TF) and signaling molecules, as well as identification and characterization of the molecular and biochemical mechanisms they regulate during the organization of distinct hypothalamic regions, their differentiation, and circuit connectivity (reviewed by Machluf et al., 2011; Pearson and Placzek, 2013). Interestingly, some of these essential developmental factors are also expressed in the mature hypothalamus (Bardet et al., 2008; Tolson et al., 2010). A few studies have directly addressed the non-developmental role of these factors in the proper functioning of mature hypothalamic nuclei, suggesting that proper regulation by these factors is essential for normal activation of the hypothalamus. These findings support the hypothesis that developmental and post-developmental impairment of these components may lead to hypothalamus-related disorders, such as infertility, obesity, depression and chronic stress.

Here, we summarize some key developmental factors involved in hypothalamic development and function. The periventricular zone of the hypothalamus contains several nuclei including the paraventricular nucleus (PVN), arcuate nucleus (Arc), supra-chiasmatic nucleus, and the anterior periventricular nucleus (aPV; Szarek et al., 2010). These hypothalamic areas are well characterized for their physiological roles, allowing the investigation of both developmental and functional regulation. Hence, we have focused our review mainly on TFs expressed in this brain region. To gain better insights on the molecular mechanisms conveyed by these TFs, we mainly focused on the mouse and zebrafish genetic models, in which specific genetic perturbations have unveiled the functions of these factors. Lastly, we discuss the possible link between factors that regulate hypothalamic development to neurodevelopmental disorders that disrupt both physiological and psychological homeostasis.

## Comparative Neuroanatomy of the Hypothalamus

In recent years, several publications emphasized the importance and relevance of non-mammalian model organisms for the study of hypothalamic development with zebrafish as the prominently utilized model (reviewed in Machluf et al., 2011; Pearson and Placzek, 2013; Wircer et al., in press). The general organization of the vertebrate brain is evolutionarily conserved, however several events during tetrapod and mammalian evolution led to neuroanatomical changes (Suárez et al., 2014). Importantly, it has been shown that key genetic factors driving the patterning and specification of major hypothalamic nuclei are evolutionarily conserved (Tessmar-Raible et al., 2007). Hence, understanding the neuroanatomical homology of the various hypothalamic nuclei is important in order to integrate the information, which has been obtained from various model organisms.

**Figure 1:**
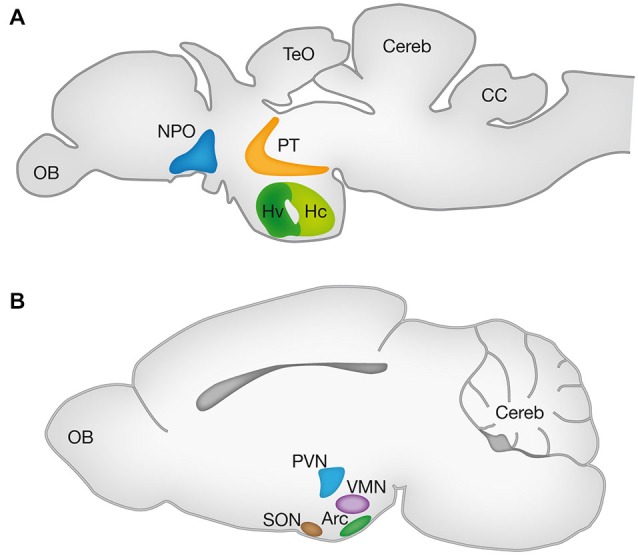
**Hypothalamic nuclei in vertebrates**. Schematic lateral view of the zebrafish **(A)** and mouse **(B)** brains representing the projected 2D anatomy of multiple sagittal planes. Color matched areas represents the presumed homology between specific hypothalamic areas of zebrafish and mouse (see text). Arc, arcuate nucleus; CC, crista cerebellaris; CCe, corpus cerebelli; Hv: ventral zone of periventricular hypothalamus; Hc, caudal zone of periventricular hypothalamus; NPO, neurosecretory preoptic area; OB, olfactory bulb; PT, posterior tuberculum; PVN, paraventricular nucleus; SON, supraoptic nucleus; TeO, tectum opticum; VMN, ventromedial nucleus.

One approach for comparative identification of the hypothalamic nuclei is based on expression patterns of mRNAs and proteins of evolutionarily conserved TFs and neuropeptides. In this regards, we will discuss findings from two prevalent genetic models namely the mouse and zebrafish, focusing on the Arc, PVN and supraoptic nuclei (SON) of the mouse hypothalamus and the ventral zone of the periventricular hypothalamus (Hv), neurosecretory preoptic area (NPO) and ventral posterior tuberculum (vPT) of the zebrafish. Schematic localization of these nuclei in the adult brains of these animals is illustrated in Figure 1.

Several studies have demonstrated that the Hv of the zebrafish (also known as nucleus lateralis tuberis; NLT) and the mammalian Arc are homologous as both nuclei express Neurokinin B (Ramaswamy et al., 2010; Biran et al., 2012; Ogawa et al., 2012), Kisspeptin (Ramaswamy et al., 2010; Servili et al., 2011; Ogawa et al., 2012), GHRH (Farhy and Veldhuis, 2004; Castro et al., 2009), αMSH and AgRP (Forlano and Cone, 2007; Guzmán-Ruiz et al., 2014). The NPO of the fish and its homologous mammalian PVN were shown to express oxytocin (OXT; Wang and Lufkin, 2000; Goodson et al., 2003; Unger and Glasgow, 2003; Löhr et al., 2009; Gutnick et al., 2011; Fernandes et al., 2013; Herget et al., 2014), Arginine vasopressin (AVP; Wang and Lufkin, 2000; Eaton et al., 2008; Löhr et al., 2009; Fernandes et al., 2013; Herget et al., 2014), Corticotropin-releasing hormone (CRH; Wang and Lufkin, 2000; Löhr et al., 2009; Amir-Zilberstein et al., 2012; Fernandes et al., 2013; Herget et al., 2014) and Somatostatin (SST; Wang and Lufkin, 2000; Blechman et al., 2007; Russek-Blum et al., 2008; Löhr et al., 2009; Fernandes et al., 2013; Herget et al., 2014).

**Figure 2:**
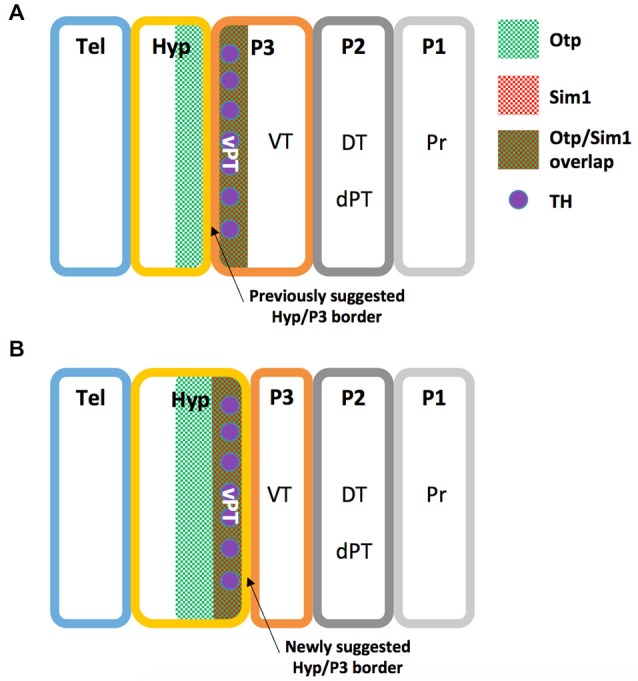
**Revised prosomere subdivision of the zebrafish forebrain. (A)** The previously suggested zebrafish prosomeric model (see text). **(B)** The newly suggested zebrafish prosomeric model based on Otp and Sim1 expression patterns. In this model, the ventral boundary between the hypothalamus and prosomere 3 is shifted to the caudal limit of Sim1 and Otp domains, while the ventral posterior tuberculum (vPT), is included in the zebrafish hypothalamus. DT, dorsal thalamus; dPT, dorsal part of the posterior tuberculum; Hyp, hypothalamus; P1, prosomere 1; P2, prosomere 2; P3, prosomere 3; Pr, pretectum; Tel, telencephalon; TH, Tyrosine hydroxylase positive neurons; vPT, ventral part of the posterior tuberculum; VT, ventral thalamus.

A recent study illustrates the high homology between the zebrafish NPO and the mammalian PVN by both peptidergic and specific NPO TFs (Herget et al., 2014). However, no piscine hypothalamic nucleus is recognized as homologous to the mammalian SON, a key hypothalamic nucleus expressing the neurohypophyseal hormones OXT and AVP (Wircer et al., in press). Moreover, the piscine NPO was recently suggested as the common evolutionary ancestor of the vertebrate magnocellular neuronal cluster, which later anatomically partitions to generate the PVN and SON in mammals (Gutnick et al., 2011; Herget et al., 2014; Knobloch and Grinevich, 2014). However, since mammalian PVN and SON neurons differ in their origin from the preoptic area neurons (Altman and Bayer, 1978a,b; Markakis, 2002), this hypothesis should be carefully considered. Interestingly, the PT in fish is another brain region that is not considered a classical hypothalamic region (Wullimann and Rink, 2001, 2002), but a careful look at the literature might suggest otherwise. Firstly, the periventricular zone of PT (vPT) was shown to express neuropeptides which are characteristic of the mammalian periventricular hypothalamus such as AVP (Wang and Lufkin, 2000; Hatae et al., 2001; Goodson et al., 2003; Eaton et al., 2008; Fernandes et al., 2013), CRH (Wang and Lufkin, 2000; Amir-Zilberstein et al., 2012; Fernandes et al., 2013) and Neurokinin B (Hatae et al., 2001; Biran et al., 2012). Secondly, catecholaminergic (tyrosine hydroxylase expressing) cells of the vPT were suggested to be homologous to hypothalamic group A11 of dopaminergic cells (Ryu et al., 2007; Löhr et al., 2009; Filippi et al., 2014). Notably, Puelles and Rubenstein define the boundary between the caudal hypothalamus and diencephalic prosomere 3 in rodents by the expression of several genes, including the TFs Single minded (Sim1) and Orthopedia (Otp), which are expressed exclusively in the hypothalamus (Puelles and Rubenstein, 1993, 2003). As both Sim1 and Otp are expessed in the zebrafish vPT (Borodovsky et al., 2009; Löhr et al., 2009; Fernandes et al., 2013) we propose a revised prosomere subdivision of the zebrafish forebrain (Figure 2), in which the boundary between the caudal hypothalamus and prosomere 3 is shifted to the caudal limit of Sim1 and Otp domains, so that the vPT is regarded as part of the teleostian hypothalamus. Future comparative gene expression analyses, fate-mapping experiments and other comparative anatomy experiments are required to further establish this refined model.

## Transcription Factors Regulating the Development of the Vertebrate Hypothalamus

The hypothalamus contains anatomical partitioning and its various neuronal cell populations form elaborate connectivity with virtually all parts of the nervous system. This raises many questions regarding the mechanisms that underlie the development of hypothalamic brain nuclei and the specification of the neuronal populations that inhabit the hypothalamus.

### Otp−

The homedomain-containing TF Otp is well conserved across species. The deduced amino acid sequence of the homeodomain of the human protein is 99% homologous to the mouse Otp, and demonstrates high degree of conservation when compared to sea urchin, drosophila (Lin et al., 1999) and planaria (Umesono et al., 1997). Additionally, the existence of several evolutionarily conserved non-coding sequences (ECR) was recently demonstrated in the Otp promoter by Gutierrez-Triana et al. (2014). Using zebrafish as their model, the authors have further demonstrated that OtpaECR6 specifically regulates the expression of Otp in the NPO of the zebrafish (Gutierrez-Triana et al., 2014). Taken together, this conservation suggests an evolutionarily conserved functional role for Otp in vertebrates. Otp is expressed in conserved hypothalamic domains (Simeone et al., 1994; Bardet et al., 2008; Del Giacco et al., 2008), where it plays an important role in the differentiation of several neurohormone—secreting nuclei including the aPV, PVN, SON, Arc and ventromedial nucleus (VMN; Acampora et al., 1999; Blechman et al., 2007; Eaton et al., 2008). In the zebrafish, the expression of Otp in the NPO and PT is regulated by the zinc-finger-containing TF Fezf2 (Blechman et al., 2007; Machluf et al., 2011; Yang et al., 2012; Wolf and Ryu, 2013). Non-hypothalamic expression of Otp is also detected in the medial amygdaloid nucleus (MeA), hindbrain and spinal cord (Simeone et al., 1994; Acampora et al., 1999). Importantly, Otp positive neurons inhabiting the murine MeA are of diencephalic origin. These neurons are generated in the hypothalamus and migrate during brain development through the diencephalic-mesencephalic junction into their final position in the MeA (García-Moreno et al., 2010).

It has been demonstrated that Otp is crucial for proper development of diencephalic dopaminergic neurons in zebrafish and mouse. Zebrafish embryos lacking the Otpa protein are devoid of dopaminergic neurons in the hypothalamus and the PT while overexpression of Otp can induce ectopic expression of dopaminergic markers, such as TH and dopamine transporter indicating that Otp can instruct dopaminergic identity (Ryu et al., 2007; Fernandes et al., 2013). Otp cooperates with another TF, Sim1 (see below) to regulate the expression of TH, CRH, TRH, SST, OXT and AVP in the NPO and PT of zebrafish (Eaton et al., 2008; Borodovsky et al., 2009; Löhr et al., 2009; Fernandes et al., 2013).

As in the zebrafish, Otp^−/−^ mouse embryos lack diencephalic dopaminergic neurons of the diencephalospinal dopaminergic system (Acampora et al., 1999; Wang and Lufkin, 2000). Homozygous Otp^−/−^ mutant mice die soon after birth and display progressive impairment of crucial neuroendocrine developmental events such as reduced cell proliferation, abnormal cell migration and failure in terminal differentiation of neurons of the PVN, SON, and Arc (Acampora et al., 1999). Further analysis of Otp mutants revealed that Otp contributes to the patterning of the hypothalamus and preoptic region, and is required for differentiation of specific OXT, AVP, CRH and SST expressing cells (Acampora et al., 1999; Wang and Lufkin, 2000). Analysis of Otp^−/−^ mouse embryos demonstrated that Otp-expressing cells fail to properly migrate from the hypothalamus to the amygdaloidal complex, leading to structural impairments in several amygdaloidal nuclei (García-Moreno et al., 2010). This data suggest that in addition to its role as a developmental regulator of several neuroendocrine lineages, Otp is also an important regulator of migratory processes of other neuronal populations.

### Sim1 and Arnt2−

Sim1 and Arnt2 are two PAS (PER-Arnt-Sim) containing TFs belonging to the large basic loop-helix-loop (bHLH) family of TFs (Ema et al., 1996; Fan et al., 1996). Sim proteins present considerable sequence divergence from the* Drosophila melanogaster* protein. Sim form a heterodimeric protein complex with the aryl hydrocarbon receptor nuclear translocator (Arnt) to activate or repress their target genes containing the so called central midline enhancer (CME) or hypoxic response element (HRE) repeats (Moffett and Pelletier, 2000; Woods et al., 2008). Data from mice and zebrafish suggest that the heterodimeric Sim1-Arnt2 complex regulates hypothalamic differentiation *in vivo* (Michaud et al., 2000; Löhr et al., 2009).

Sim1 null and Arnt2 null mice die shortly after birth and analysis of newborn brains indicates the lack of the hypothalamic aPV, PVN, and SON nuclei, phenocopying the Otp null phenotype (Michaud et al., 1998; Keith et al., 2001). However, Otp null mice show a dramatic decrease of almost 30% in brain size (Wang and Lufkin, 2000) while Sim1, Sim2 and Arnt2 null mice display developmental impairments that are correlated with deficits in neuronal migration and differentiation (Michaud et al., 1998, 2000; Goshu et al., 2004).

Sim1, Arnt2 and Otp function along parallel pathways, as they are all required for Sim2 expression in the PVN for the differentiation of the neurons secreting TRH, and in the aPV for the differentiation of the neurons that secrete SST. In the PVN and SON nuclei, these TFs are required for the maintenance of Brn2 expression, a POU domain TF necessary for the development of OXT, AVP, and CRH producing neurons (Schonemann et al., 1995; Michaud et al., 1998, 2000; Acampora et al., 1999; Wang and Lufkin, 2000; Keith et al., 2001; Goshu et al., 2004).

### Steroidogenic Factor 1 (SF-1)

The VMN of the hypothalamus is involved in the regulation of many homeostatic functions, such as the maintenance of energy balance, sexual behavior, anxiety and circadian rhythms (McClellan et al., 2006; Cheung et al., 2013). However, the development of the VMN is less characterized in comparison to the highly studied periventricular zone of the hypothalamus. The finding that SF-1 null mice lack a recognizable hypothalamic VMN, now allows the investigation of neuroanatomical and functional development of this non-peptidergic hypothalamic nucleus. SF-1 is an orphan nuclear receptor (also known as adrenal 4-binding protein; Ad4BP or Nr5a1). It was originally identified as a transcriptional regulator of cytochrome P450 steroid hydroxylases enzymes that are involved in the biosynthesis of steroid hormones (Omura and Morohashi, 1995; Parker and Schimmer, 1995). It is highly conserved both in structure and function throughout evolution (Luo et al., 1994; Achermann et al., 1999; Takase et al., 2000; Allen and Spradling, 2008). SF-1 is expressed in steroidogenic cells in the gonads, adrenal cortex and spleen as well as in the VMN and the anterior pituitary. At E11.0 SF-1 is expressed by diencephalic cells which will form the VMN (Ikeda et al., 2001; Tran et al., 2003). It is expressed as early as E9.0 during embryonic development of the mouse by cells which at later stages, will form the gonads in both sexes. At around E12.5, it shows sexual di-morphism with a higher expression in testes than ovaries (Luo et al., 1994; Ikeda et al., 2001; Sekido and Lovell-Badge, 2008). Notably, SF-1 is important for endocrine cell-fate specification (Lee et al., 2011). Mice lacking SF-1 expression die shortly after birth of adrenocortical insufficiency (Luo et al., 1994; Parker et al., 2002). These knock-out mice do not develop an adrenal gland or gonads. Similar defects in adrenal and gonadal development are also apparent in humans who carry mutations in the *sf-1* gene (Luo et al., 1994; Achermann et al., 1999). Disruption of SF-1 also results in structural and neuronal connectivity alterations in the VMN (Shinoda et al., 1995; Tran et al., 2003; Zhao et al., 2008; Cheung et al., 2013). Yet it seems that in the absence of functional SF-1 the initial migration and proliferation of the neuronal precursors remains unaffected, whereas it is important for terminal differentiation of these hypothalamic neurons (Tran et al., 2003). Interestingly, *in vitro* analysis of various promoters reveals potential SF-1 binding sites in the *fezf1, A2bp1, Nkx2-2*, *Slitrk1* and *Slitrk5* genes that are involved in neuronal differentiation and patterning (Kurrasch et al., 2007).

## Receptors and Ligands Regulating Hypothalamic Development

The migration of a specific neuronal type into the hypothalamus, settlement of specific neuronal populations in distinct hypothalamic nuclei as well as their proper connectivity with a variety of target sites further requires extrinsic signals such as growth factors, neuropeptides and their receptors. These act concomitantly with the aforementioned intrinsic TFs to regulate the above processes. Moreover, several recent findings indicate that some neuropeptides, are involved in the development of neural circuits in which they function in the mature hypothalamus. This suggests that at least some neuropeptides act as “developmental autoregulators”. This section demonstrates the importance of these extrinsic factors for the patterning and assembly of neurocircuits in the developing hypothalamus.

### Extrinsic Developmental Factors

Sonic hedgehog (SHH) which is probably the most characterized morphogen was shown to be crucial for the growth and axial patterning of the hypothalamus (Mathieu et al., 2002; Szabó et al., 2009; Alvarez-Bolado et al., 2012). Moreover, SHH was shown to directly regulate the expression of its cognate receptor Patched 1 (PTCH1) through which it probably signals to promote anterior-dorsal hypothalamic fate and to antagonize Nodal activity in the development of the posterior-ventral hypothalamus (Concordet et al., 1996; Koudijs et al., 2008; Szabó et al., 2009). SHH cooperates with Nodal in the maintenance of the anterior-dorsal hypothalamus (Mathieu et al., 2002) and with bone morphogenetic proteins (BMPs) to drive hypothalamic dopaminergic neuronal specification (Ohyama et al., 2005). Genetic disruption of BMP-receptor1a from olig1 cell lineage led to a decreased number of dopaminergic and proopiomelanocortin (POMC) neurons and increased neuropeptide Y (NPY) neurons in the Arc leading to hypophagic phenotype (Peng et al., 2012). Although this genetic manipulation led to increased expression of the orexigenic AgRP, there was a profound impairment in their fiber numbers (Peng et al., 2012). As both Nodal and BMP are members of the transforming growth factor-β (TGF-β) superfamily, this data suggests interaction between SHH and TGF-β signaling pathways during hypothalamic development.

Wnt and its receptor Frizzled are also important regulators of hypothalamic differentiation and Wnt signaling components such as wnt8b, Frizzled8a and Lef1 were shown to regulate the patterning, neurogenesis and differentiation of posterior hypothalamic cells of zebrafish (Kim et al., 2002; Lee et al., 2006; Russek-Blum et al., 2008). In addition, other members of the Wnt cascade were identified in the Arc of the mouse (Benzler et al., 2013). As the expression of SHH, Nodal, BMP and Wnt is maintained in the mature brain it would be interesting to see whether these molecules are released in a synaptic manner, or maintain their activity via a diffusion mechanism in the adult hypothalamus.

### Peptides and Their Receptors

PAC1 (A.K.A ADCYAP1R1) which is the most specific (i.e., high-affinity) receptor for the pleiotropic neuropeptide pituitary adenylate cyclase-activating polypeptide (PACAP) was shown to regulate the development of zebrafish dopaminergic and OXT neurons by controlling the rate of Otp protein synthesis (Blechman et al., 2007). The anorexigenic peptide Leptin is known to regulate metabolic related homeostatic functions in the hypothalamus through its cognate Leptin receptor (LepR; reviewed by Münzberg and Morrison, 2015). Interestingly, leptin was shown to directly regulate developmental neurite formation of Arc neurons (Bouret et al., 2004). It regulates the neural projections of both orexigenic NPY and anorexigenic POMC neurons (Bouret et al., 2012). This seemingly contradictory effect in which leptin regulates the plasticity of two neuronal populations with opposing effects on energy balance, could be partly explained by the developmental switch in leptin regulation of NPY neurons during the postnatal weaning maturational period (Baquero et al., 2014).

The roles of PACAP, Leptin and their receptors in regulating adult brain and hypothalamic functions were thoroughly investigated in the last decades (reviewed by Blechman and Levkowitz, 2013; Matsuda et al., 2013; Münzberg and Morrison, 2015). The above examples show that these peptidergic systems, which were previously considered as regulators of mature functions, also play a role in the developing hypothalamus. Thus, at least some neuropeptides regulate not only the function but also the development of the systems they control.

## Adult Functions of Developmental Factors

Several key developmental factors have been found to be expressed in the mature hypothalamus, however, their post-developmental roles in homeostatic regulation remain elusive. This section presents the current data regarding the adult function of the developmental-related TFs described in previous sections of this manuscript.

### Otp−

**Figure 3:**
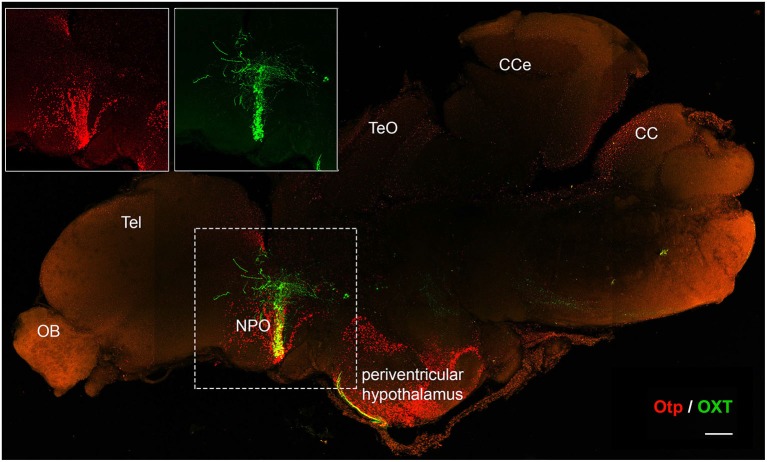
**Otp expression is maintained in the adult brain**. Immunofluorescence staining of Otp (red) and oxytocin (OXT) EGFP reporter (Blechman et al., 2011) (green) in a two year-old zebrafish brain. The image shows tiled maximum intensity projection of a mid-sagital section (150 µm). Insets display separate single channel images of Otp and OXT in the NPO. CC, crista cerebellaris; CCe, corpus cerebelli; NPO, neurosecretory preoptic area; OB, olfactory bulb; Tel, telencephalon; TeO, tectum opticum. Scale bar, 200 µm.

It has been suggested that a common ancestor of all ray-finned fish experienced a whole genome duplication event early in evolution, about 350 million years ago (Amores et al., 1998; Vandepoele et al., 2004; Dehal and Boore, 2005; Brunet et al., 2006). As a result, the zebrafish genome contains two Otp genes, *otpa* and *otpb*, which share high sequence and expression-pattern homology and present partial redundancy in function (Ryu et al., 2007; Fernandes et al., 2013). Otp expression is maintained in the mature hypothalamus of mouse and zebrafish (Amir-Zilberstein et al., 2012; Herget et al., 2014; Figure 3). This suggests that alongside its crucial role in embryonic development, Otp is also involved in adult hypothalamic function. While Otp-null mice die shortly after birth and conditional allele for the gene currently does not exist, the duplication and partitioning of the gene in zebrafish allows both *otpa*^−/−^ or *otpb*^−/−^ fish to survive into adulthood (Ryu et al., 2007; Fernandes et al., 2013). This enables the investigation of Otp’s role in post-developmental and adult hypothalamic function. Data acquired from adult Otp-null zebrafish, point to the homeostatic activities Otp regulates in adult brains. Adult Otpa-null zebrafish demonstrated impaired anxiety-like behavior compared with their wild-type siblings in response to “novel-tank” diving test. Mutant fish spent more time in the top zone of the tank during the first 2 min of the assay suggesting that Otp is involved in the regulation of novelty related stressors (Amir-Zilberstein et al., 2012; Blechman and Levkowitz, 2013). Otp mutant zebrafish also display deficits in the molecular response of the hypothalamo-pituitary-adrenal (HPA) axis to stressors. These include the regulation of CRH transcription and cortisol response. Thus, Otp directly regulates CRH gene expression in zebrafish and mouse (Amir-Zilberstein et al., 2012). Moreover, Otp indirectly regulates the alternative splicing of the aforementioned PAC1 receptor during stress adaptation phase that follows various homeostatic challenges (Amir-Zilberstein et al., 2012). Thus in addition to its role in hypothalamic development, Otp may act as a cellular sensor, which mediate between a given homeostatic challenge (“input”) and the following hypothalamic hormonal response (“output”). This assumption is reinforced by the finding that Otp-positive neurons in the zebrafish NPO modulate the visual motor response through the regulation of the melanopsin 4a (opn4a) receptor. This apparent Otp-mediated “non-visual” deep brain light-sensing system indicates that Otp neurons serve as an extra-ocular photoreception center in dark photokinesis behavior (Fernandes et al., 2012).

Taken together with its known role in regulating several types of neuropeptidergic neurons in the NPO/PVN, these findings suggest that Otp orchestrates the physiological response to environmental challenges. Given the importance of Otp in hypothalamic function, comprehensive research regarding its expression patterns and stress-induced molecular targets in response to physiological and psychological challenges, is further required.

### Sim1−

Sim1 displays haploinsufficiency unveiling its function in metabolic regulation. Sim1^+/−^ mice possess a hypocellular PVN and are hyperphagic and obese with increased linear growth, hyperinsulinemia and hyperleptinemia, mainly under high fat diet conditions (Michaud et al., 2001; Holder et al., 2004; Kublaoui et al., 2006). Sim1 heterozygotes display normal energy expenditure, and treatment with the melanocortin receptor agonist, MTII, increases energy expenditure in both WT and Sim1 heterozygous mice (Kublaoui et al., 2006). This phenotype is further supported by data from humans with balanced chromosomal translocations or genomic mutations, which interrupts the Sim1 gene. In spite of their normal basal metabolic rate, these subjects usually suffer from early onset obesity, increased food intake, and display evidence of neurobehavioral abnormalities (Holder et al., 2000; Ramachandrappa et al., 2013).

Sim1^+/−^ mice display reduced expression of several hypothalamic neuropeptides such as TRH, CRH, AVP, and SST (Kublaoui et al., 2008). In line with their low OXT levels, Sim1 heterozygotes also show higher sensitivity to the orexigenic effect of the OXT receptor antagonist, OVT (Kublaoui et al., 2008). Furthermore, intracerebroventricular administration of OXT to Sim1^+/−^ rescues the hyperphagic phenotype and reduces the characteristic weight increase of heterozygotes. Postnatal chemical ablation of Sim1 expressing neurons leads to hyperphagic obesity and reduced expression of OXT and TRH. However, while Sim1 heterozygotes or post-developmental knockouts display normal energy expenditure, ablation of Sim1-positive neurons leads to decreased energy expenditure (Xi et al., 2012). Postnatal PVN-specific ablation of Sim1 combined with chow diet leads to a hyperphagic obesity phenotype. However, when fed with high-fat diet, the trend is reversed and these mice display reduced food intake and weight loss. Since PVN specific ablation of Sim1 neurons also leads to increased Sim1 expression in the Amygdala, the key regulating region of this phenotype remains to be determined (Xi et al., 2013).

In view of the above data, the authors suggested a model arguing that Sim1 heterozygous phenotype of obesity and hyperphagia occur due to the Sim1 regulatory effect on OXT which is also severely depleted in Sim1 heterozygotes and melanocortin recptor-4 (Mc4r), both of which are known to function in appetite regulation (Kublaoui et al., 2006, 2008; Tolson et al., 2010). The accumulating data further support the fact that at least some of the phenotypes correlated with lack of Sim1 are caused by the perturbation of its mature brain functioning rather than from developmental impairments.

### SF-1−

SF-1 positive neurons are directly involved in the regulation of body weight in the mature brain. This function is mediated, among others, by their responsiveness to the hormone leptin. Hence, specific deletion of leptin receptor in SF-1 neurons results in defects in the ability to maintain normal energy homeostasis and these mice display increased body weight (Dhillon et al., 2006). In addition, there are some indications that SF-1 may directly regulate the expression of the brain-derived neurotrophic factor (BDNF; Tran et al., 2003, 2006), a growth factor which is involved in energy balance (Xu et al., 2003). Yet, it is not clear whether SF-1 indeed regulates BDNF *in vivo*, and if so—what the physiological significance of this regulation is (Dhillon et al., 2006).

Alongside its role in the maintenance of energy balance SF-1 may be involved in the modulation of the HPA axis in response to stress. SF-1 heterozygous mice develop normal VMN and pituitary, but have defects in their adrenal and in their stress response. These mice display abnormal circulating levels of ACTH and corticosterone during the day and following stressful challenges (Bland et al., 2000). In addition, mice with CNS-specific SF-1 knock-out display anxiety-like behaviors in response to various environmental challenges (Zhao et al., 2008). Beyond the developmental defects that disrupt the normal stress response, *in vitro* experiments indicate the involvement of SF-1 in the direct regulation of the CRH receptor-2 gene, and in SF-1 knock-out mice there is a marked reduction in CRH receptor expression in the VMN (Zhao et al., 2008).

## Concluding Remarks

The hypothalamus regulates brain and body functions by controlling the activity of a variety of neuropeptide-containing cell types. By doing so, it allows vertebrates to orchestrate multiple homeostatic processes in order to adapt to the ever-changing environment, thereby maintaining the organism’s survival and reproduction. Therefore, mechanistic understanding of the patterning and differentiation of the hypothalamus may further shed light on the function of the mature hypothalamus.

The increased use of zebrafish as a model organism allows the combination of powerful genetic tools with high-resolution imaging techniques to advance our knowledge regarding the molecular pathways governing hypothalamic development. One obvious advantage in utilizing both mammalian and non-mammalian models is the ability to gain wider knowledge on evolutionarily conserved regulatory pathways. However, a prerequisite for practical use of the accumulating scientific knowledge is to understand the neuroanatomical organization of the hypothalamus in the key models.

Although the knowledge regarding nucleus-specific markers and TFs continue to expand, many hypothalamic nuclei remain “uncharted”. Thus, the identification of new regulators of hypothalamic development and function is of fundamental importance. In this regards, the introduction of innovative genome editing methodologies, such as the transcription activator-like effector nuclease (TALEN) or the clustered regularly interspaced short palindromic repeats (CRISPR) genome editing methods now allow efficient genetic manipulation and analysis of new candidate genes (Doudna and Charpentier, 2014; Wright et al., 2014).

Recent findings have demonstrated that some genetic pathways that are involved in hypothalamic development, also play a role in mature hypothalamic functions (summarized in Table 1). Thus, key developmental factors maintain their expression in the mature hypothalamus. As developmental perturbation of these genes might lead to lethality, the use of conditional and inducible knockout models is necessary. For example, Sim1 post-developmental knockouts demonstrate the valuable scientific knowledge that can be achieved when using such models, unveiling the highly important role of Sim1 in feeding and metabolic regulation in the mature hypothalamus.

**Table 1:** **Key functions demonstrated for transcription factors expressed in both developing and mature hypothalamus**.

Factor	Developmental function	Function in adult
**Otp**	Differentiation of the neurons of the aPV, PVN, and SON (Blechman et al., 2007; Eaton et al., 2008) Development of diencephalic dopaminergic neurons in zebrafish and mouse (Ryu et al., 2007) Required for expression of TH, CRH, TRH, SST, OT and AVP in the dorsal preoptic area and posterior tuberculum of zebrafish (Eaton et al., 2008; Löhr et al., 2009; Fernandes et al., 2013)	Regulation of CRH expression (Amir-Zilberstein et al., 2012) Maintenance of opn4a in the aPO, thus regulating dark photokinesis (Fernandes et al., 2012)
**Sim1**	Differentiation of TRH neurons in the PVN and SST neurons in the aPV (Schonemann et al., 1995; Michaud et al., 1998, 2000; Acampora et al., 1999; Wang and Lufkin, 2000; Keith et al., 2001; Goshu et al., 2004); Maintenance of Brn2 expression in the PVN and SON nuclei (Schonemann et al., 1995; Michaud et al., 1998, 2000; Acampora et al., 1999; Wang and Lufkin, 2000; Keith et al., 2001; Goshu et al., 2004) Together with Otp, required for expression of TH, CRH, TRH, SST, OT and AVP in the dorsal preoptic area and posterior tuberculum of zebrafish (Eaton et al., 2008; Löhr et al., 2009; Fernandes et al., 2013)	Control of OXT expression to affect appetite regulation (Kublaoui et al., 2006, 2008; Tolson et al., 2010)
**SF-1**	Terminal differentiation of VMN neurons (Tran et al., 2003)	Involvement in energy balance via maintenance of leptin-receptor (Dhillon et al., 2006) Regulation of BDNF expression (Xu et al., 2003) Modulation of the peripheral HPA axis in response to stress (Bland et al., 2000) Direct regulation of *crhr2* expression *in vitro* (Zhao et al., 2008)

## Conflict of Interest Statement

The authors declare that the research was conducted in the absence of any commercial or financial relationships that could be construed as a potential conflict of interest.

## References

[B1] Acampora D. Postiglione M. P. Avantaggiato V. Di Bonito M. Vaccarino F. M. Michaud J.. (1999). Progressive impairment of developing neuroendocrine cell lineages in the hypothalamus of mice lacking the Orthopedia gene. Genes Dev. 13, 2787–2800. 10.1101/gad.13.21.2787 10557207 PMC317121

[B2] Achermann J. C. Ito M. Ito M. Hindmarsh P. C. Jameson J. L. (1999). A mutation in the gene encoding steroidogenic factor-1 causes XY sex reversal and adrenal failure in humans. Nat. Genet. 22, 125–126. 10.1038/9629 10369247

[B3] Allen A. K. Spradling A. C. (2008). The Sf1-related nuclear hormone receptor Hr39 regulates *Drosophila* female reproductive tract development and function. Development 135, 311–321. 10.1242/dev.015156 18077584

[B4] Altman J. Bayer S. A. (1978a). Development of the diencephalon in the rat. I. Autoradiographic study of the time of origin and settling patterns of neurons of the hypothalamus. J. Comp. Neurol. 182, 945–971. 10.1002/cne.901820511 103939

[B5] Altman J. Bayer S. A. (1978b). Development of the diencephalon in the rat. II. Correlation of the embryonic development of the hypothalamus with the time of origin of its neurons. J. Comp. Neurol. 182, 973–993. 10.1002/cne.901820512 103940

[B6] Alvarez-Bolado G. Paul F. A. Blaess S. (2012). Sonic hedgehog lineage in the mouse hypothalamus: from progenitor domains to hypothalamic regions. Neural Dev. 7:4. 10.1186/1749-8104-7-4 22264356 PMC3292819

[B7] Amir-Zilberstein L. Blechman J. Sztainberg Y. Norton W. H. Reuveny A. Borodovsky N.. (2012). Homeodomain protein otp and activity-dependent splicing modulate neuronal adaptation to stress. Neuron 73, 279–291. 10.1016/j.neuron.2011.11.019 22284183 PMC4387198

[B8] Amores A. Force A. Yan Y. L. Joly L. Amemiya C. Fritz A.. (1998). Zebrafish hox clusters and vertebrate genome evolution. Science 282, 1711–1714. 10.1126/science.282.5394.1711 9831563

[B9] Baquero A. F. de Solis A. J. Lindsley S. R. Kirigiti M. A. Smith M. S. Cowley M. A.. (2014). Developmental switch of leptin signaling in arcuate nucleus neurons. J. Neurosci. 34, 9982–9994. 10.1523/JNEUROSCI.0933-14.2014 25057200 PMC4107412

[B10] Bardet S. M. Martinez-de-la-Torre M. Northcutt R. G. Rubenstein J. L. Puelles L. (2008). Conserved pattern of OTP-positive cells in the paraventricular nucleus and other hypothalamic sites of tetrapods. Brain Res. Bull. 75, 231–235. 10.1016/j.brainresbull.2007.10.037 18331876

[B11] Benzler J. Andrews Z. B. Pracht C. Stöhr S. Shepherd P. R. Grattan D. R.. (2013). Hypothalamic WNT signalling is impaired during obesity and reinstated by leptin treatment in male mice. Endocrinology 154, 4737–4745. 10.1210/en.2013-1746 24105484

[B12] Biran J. Palevitch O. Ben-Dor S. Levavi-Sivan B. (2012). Neurokinin Bs and neurokinin B receptors in zebrafish-potential role in controlling fish reproduction. Proc. Natl. Acad. Sci. U S A 109, 10269–10274. 10.1073/pnas.1119165109 22689988 PMC3387093

[B13] Bland M. L. Jamieson C. A. Akana S. F. Bornstein S. R. Eisenhofer G. Dallman M. F.. (2000). Haploinsufficiency of steroidogenic factor-1 in mice disrupts adrenal development leading to an impaired stress response. Proc. Natl. Acad. Sci. U S A 97, 14488–14493. 10.1073/pnas.97.26.14488 11121051 PMC18946

[B113] Blechman J. Amir-Zilberstein L. Gutnick A. Ben-Dor S. Levkowitz G. (2011). The metabolic regulator PGC-1α directly controls the expression of the hypothalamic neuropeptide oxytocin. J. Neurosci. 31, 14835–14840. 10.1523/JNEUROSCI.1798-11.2011 22016516 PMC6623572

[B14] Blechman J. Borodovsky N. Eisenberg M. Nabel-Rosen H. Grimm J. Levkowitz G. (2007). Specification of hypothalamic neurons by dual regulation of the homeodomain protein Orthopedia. Development 134, 4417–4426. 10.1242/dev.011262 18003738

[B15] Blechman J. Levkowitz G. (2013). Alternative Splicing of the Pituitary Adenylate Cyclase-Activating Polypeptide receptor PAC1: mechanisms of fine tuning of brain activity. Front. Endocrinol. (Lausanne) 4:55. 10.3389/fendo.2013.00055 23734144 PMC3659299

[B16] Borodovsky N. Ponomaryov T. Frenkel S. Levkowitz G. (2009). Neural protein Olig2 acts upstream of the transcriptional regulator Sim1 to specify diencephalic dopaminergic neurons. Dev. Dyn. 238, 826–834. 10.1002/dvdy.21894 19253397

[B17] Bouret S. G. Bates S. H. Chen S. Myers M. G. Jr. Simerly R. B. (2012). Distinct roles for specific leptin receptor signals in the development of hypothalamic feeding circuits. J. Neurosci. 32, 1244–1252. 10.1523/JNEUROSCI.2277-11.2012 22279209 PMC3567460

[B18] Bouret S. G. Draper S. J. Simerly R. B. (2004). Trophic action of leptin on hypothalamic neurons that regulate feeding. Science 304, 108–110. 10.1126/science.1095004 15064420

[B19] Brunet F. G. Roest Crollius H. Paris M. Aury J. M. Gibert P. Jaillon O.. (2006). Gene loss and evolutionary rates following whole-genome duplication in teleost fishes. Mol. Biol. Evol. 23, 1808–1816. 10.1093/molbev/msl049 16809621

[B20] Castro A. Becerra M. Manso M. J. Tello J. Sherwood N. M. Anadón R. (2009). Distribution of growth hormone-releasing hormone-like peptide: immunoreactivity in the central nervous system of the adult zebrafish (Danio rerio). J. Comp. Neurol. 513, 685–701. 10.1002/cne.21977 19235874

[B21] Cheung C. C. Kurrasch D. M. Liang J. K. Ingraham H. A. (2013). Genetic labeling of steroidogenic factor-1 (SF-1) neurons in mice reveals ventromedial nucleus of the hypothalamus (VMH) circuitry beginning at neurogenesis and development of a separate non-SF-1 neuronal cluster in the ventrolateral VMH. J. Comp. Neurol. 521, 1268–1288. 10.1002/cne.23226 22987798 PMC4304766

[B22] Concordet J. P. Lewis K. E. Moore J. W. Goodrich L. V. Johnson R. L. Scott M. P.. (1996). Spatial regulation of a zebrafish patched homologue reflects the roles of sonic hedgehog and protein kinase A in neural tube and somite patterning. Development 122, 2835–2846. 8787757 10.1242/dev.122.9.2835

[B23] Dehal P. Boore J. L. (2005). Two rounds of whole genome duplication in the ancestral vertebrate. PLoS Biol. 3:e314. 10.1371/journal.pbio.0030314 16128622 PMC1197285

[B24] Del Giacco L. Pistocchi A. Cotelli F. Fortunato A. E. Sordino P. (2008). A peek inside the neurosecretory brain through Orthopedia lenses. Dev. Dyn. 237, 2295–2303. 10.1002/dvdy.21668 18729222

[B84] de Seranno S. d’Anglemont de Tassigny X. Estrella C. Loyens A. Kasparov S. Leroy D.. (2010). Role of Estradiol in the dynamic control of Tanycyte plasticity mediated by vascular Endothelial cells in the Median Eminence. Endocrinology 151, 1760–1772. 10.1210/en.2009-0870 20133455 PMC2850227

[B25] Dhillon H. Zigman J. M. Ye C. Lee C. E. McGovern R. A. Tang V.. (2006). Leptin directly activates SF1 neurons in the VMH and this action by leptin is required for normal body-weight homeostasis. Neuron 49, 191–203. 10.1016/j.neuron.2005.12.021 16423694

[B26] Doudna J. A. Charpentier E. (2014). Genome editing. The new frontier of genome engineering with CRISPR-Cas9. Science 346:1258096. 10.1126/science.1258096 25430774

[B27] Eaton J. L. Holmqvist B. Glasgow E. (2008). Ontogeny of vasotocin-expressing cells in zebrafish: selective requirement for the transcriptional regulators orthopedia and single-minded 1 in the preoptic area. Dev. Dyn. 237, 995–1005. 10.1002/dvdy.21503 18330923

[B28] Ema M. Morita M. Ikawa S. Tanaka M. Matsuda Y. Gotoh O.. (1996). Two new members of the murine Sim gene family are transcriptional repressors and show different expression patterns during mouse embryogenesis. Mol. Cell. Biol. 16, 5865–5875. 8927054 10.1128/mcb.16.10.5865 PMC231588

[B29] Fan C. M. Kuwana E. Bulfone A. Fletcher C. F. Copeland N. G. Jenkins N. A.. (1996). Expression patterns of two murine homologs of *Drosophila* single-minded suggest possible roles in embryonic patterning and in the pathogenesis of down syndrome. Mol. Cell. Neurosci. 7, 1–16. 10.1006/mcne.1996.0001 8812055

[B30] Farhy L. S. Veldhuis J. D. (2004). Putative GH pulse renewal: periventricular somatostatinergic control of an arcuate-nuclear somatostatin and GH-releasing hormone oscillator. Am. J. Physiol. Regul. Integr. Comp. Physiol. 286, R1030–R1042. 10.1152/ajpregu.00473.2003 14988084

[B31] Fernandes A. M. Beddows E. Filippi A. Driever W. (2013). Orthopedia transcription factor otpa and otpb paralogous genes function during dopaminergic and neuroendocrine cell specification in larval zebrafish. PLoS One 8:e75002. 10.1371/journal.pone.0075002 24073233 PMC3779234

[B32] Fernandes A. M. Fero K. Arrenberg A. B. Bergeron S. A. Driever W. Burgess H. A. (2012). Deep brain photoreceptors control light-seeking behavior in zebrafish larvae. Curr. Biol. 22, 2042–2047. 10.1016/j.cub.2012.08.016 23000151 PMC3494761

[B33] Filippi A. Mueller T. Driever W. (2014). vglut2 and gad expression reveal distinct patterns of dual GABAergic versus glutamatergic cotransmitter phenotypes of dopaminergic and noradrenergic neurons in the zebrafish brain. J. Comp. Neurol. 522, 2019–2037. 10.1002/cne.23524 24374659 PMC4288968

[B34] Forlano P. M. Cone R. D. (2007). Conserved neurochemical pathways involved in hypothalamic control of energy homeostasis. J. Comp. Neurol. 505, 235–248. 10.1002/cne.21447 17879270

[B35] García-Moreno F. Pedraza M. Di Giovannantonio L. G. Di Salvio M. López-Mascaraque L. Simeone A.. (2010). A neuronal migratory pathway crossing from diencephalon to telencephalon populates amygdala nuclei. Nat. Neurosci. 13, 680–689. 10.1038/nn.2556 20495559

[B36] Goodson J. L. Evans A. K. Bass A. H. (2003). Putative isotocin distributions in sonic fish: relation to vasotocin and vocal-acoustic circuitry. J. Comp. Neurol. 462, 1–14. 10.1002/cne.10679 12761820 PMC2679688

[B37] Goshu E. Jin H. Lovejoy J. Marion J. F. Michaud J. L. Fan C. M. (2004). Sim2 contributes to neuroendocrine hormone gene expression in the anterior hypothalamus. Mol. Endocrinol. 18, 1251–1262. 10.1210/me.2003-0372 14988428

[B38] Gutierrez-Triana J. Herget U. Lichtner P. Castillo-Ramírez L. A. Ryu S. (2014). A vertebrate-conserved cis -regulatory module for targeted expression in the main hypothalamic regulatory region for the stress response. BMC Dev. Biol. 14:41. 10.1186/preaccept-1743418341322888 25427861 PMC4248439

[B39] Gutnick A. Blechman J. Kaslin J. Herwig L. Belting H. G. Affolter M.. (2011). The hypothalamic neuropeptide oxytocin is required for formation of the neurovascular interface of the pituitary. Dev. Cell 21, 642–654. 10.1016/j.devcel.2011.09.004 22014522 PMC4387193

[B40] Guzmán-Ruiz M. Saderi N. Cazarez-Márquez F. Guerrero-Vargas N. N. Basualdo M. C. Acosta-Galván G.. (2014). The suprachiasmatic nucleus changes the daily activity of the arcuate nucleus alpha-MSH neurons in male rats. Endocrinology 155, 525–535. 10.1210/en.2013-1604 24265453

[B41] Hatae T. Kawano H. Karpitskiy V. Krause J. E. Masuko S. (2001). Arginine-vasopressin neurons in the rat hypothalamus produce neurokinin B and co-express the tachykinin NK-3 receptor and angiotensin II type 1 receptor. Arch. Histol. Cytol. 64, 37–44. 10.1679/aohc.64.37 11310503

[B42] Herget U. Wolf A. Wullimann M. F. Ryu S. (2014). Molecular neuroanatomy and chemoarchitecture of the neurosecretory preoptic-hypothalamic area in zebrafish larvae. J. Comp. Neurol. 522, 1542–1564. 10.1002/cne.23480 24127437

[B43] Holder J. L. Jr. Butte N. F. Zinn A. R. (2000). Profound obesity associated with a balanced translocation that disrupts the SIM1 gene. Hum. Mol. Genet. 9, 101–108. 10.1093/hmg/9.1.101 10587584

[B44] Holder J. L. Jr. Zhang L. Kublaoui B. M. Dileone R. J. Oz O. K. Bair C. H.. (2004). Sim1 gene dosage modulates the homeostatic feeding response to increased dietary fat in mice. Am. J. Physiol. Endocrinol. Metab. 287, E105–E113. 10.1152/ajpendo.00446.2003 14982752

[B45] Ikeda Y. Takeda Y. Shikayama T. Mukai T. Hisano S. Morohashi K. I. (2001). Comparative localization of Dax-1 and Ad4BP/SF-1 during development of the hypothalamic-pituitary-gonadal axis suggests their closely related and distinct functions. Dev. Dyn. 220, 363–376. 10.1002/dvdy.1116 11307169

[B46] Keith B. Adelman D. M. Simon M. C. (2001). Targeted mutation of the murine arylhydrocarbon receptor nuclear translocator 2 (Arnt2) gene reveals partial redundancy with Arnt. Proc. Natl. Acad. Sci. U S A 98, 6692–6697. 10.1073/pnas.121494298 11381139 PMC34414

[B47] Kim S. H. Shin J. Park H. C. Yeo S. Y. Hong S. K. Han S.. (2002). Specification of an anterior neuroectoderm patterning by Frizzled8a-mediated Wnt8b signalling during late gastrulation in zebrafish. Development 129, 4443–4455. 12223403 10.1242/dev.129.19.4443

[B48] Knobloch H. S. Grinevich V. (2014). Evolution of oxytocin pathways in the brain of vertebrates. Front. Behav. Neurosci. 8:31. 10.3389/fnbeh.2014.00031 24592219 PMC3924577

[B49] Koudijs M. J. den Broeder M. J. Groot E. van Eeden F. J. (2008). Genetic analysis of the two zebrafish patched homologues identifies novel roles for the hedgehog signaling pathway. BMC Dev. Biol. 8:15. 10.1186/1471-213X-8-15 18284698 PMC2275722

[B50] Kublaoui B. M. Gemelli T. Tolson K. P. Wang Y. Zinn A. R. (2008). Oxytocin deficiency mediates hyperphagic obesity of Sim1 haploinsufficient mice. Mol. Endocrinol. 22, 1723–1734. 10.1210/me.2008-0067 18451093 PMC2453606

[B51] Kublaoui B. M. Holder J. L. Jr. Gemelli T. Zinn A. R. (2006). Sim1 haploinsufficiency impairs melanocortin-mediated anorexia and activation of paraventricular nucleus neurons. Mol. Endocrinol. 20, 2483–2492. 10.1210/me.2005-0483 16728530

[B52] Kurrasch D. M. Cheung C. C. Lee F. Y. Tran P. V. Hata K. Ingraham H. A. (2007). The neonatal ventromedial hypothalamus transcriptome reveals novel markers with spatially distinct patterning. J. Neurosci. 27, 13624–13634. 10.1523/jneurosci.2858-07.2007 18077674 PMC6673626

[B53] Lee F. Y. Faivre E. J. Suzawa M. Lontok E. Ebert D. Cai F.. (2011). Eliminating SF-1 (NR5A1) sumoylation *in vivo* results in ectopic hedgehog signaling and disruption of endocrine development. Dev. Cell 21, 315–327. 10.1016/j.devcel.2011.06.028 21820362 PMC3157481

[B54] Lee J. E. Wu S. F. Goering L. M. Dorsky R. I. (2006). Canonical Wnt signaling through Lef1 is required for hypothalamic neurogenesis. Development 133, 4451–4461. 10.1242/dev.02613 17050627

[B55] Lin X. State M. W. Vaccarino F. M. Greally J. Hass M. Leckman J. F. (1999). Identification, chromosomal assignment and expression analysis of the human homeodomain-containing gene Orthopedia (OTP). Genomics 60, 96–104. 10.1006/geno.1999.5882 10458915

[B56] Löhr H. Ryu S. Driever W. (2009). Zebrafish diencephalic A11-related dopaminergic neurons share a conserved transcriptional network with neuroendocrine cell lineages. Development 136, 1007–1017. 10.1242/dev.033878 19234064

[B57] Luo X. Ikeda Y. Parker K. L. (1994). A cell-specific nuclear receptor is essential for adrenal and gonadal development and sexual differentiation. Cell 77, 481–490. 10.1016/0092-8674(94)90211-9 8187173

[B58] Machluf Y. Gutnick A. Levkowitz G. (2011). Development of the zebrafish hypothalamus. Ann. N Y Acad. Sci. 1220, 93–105. 10.1111/j.1749-6632.2010.05945.x 21388407

[B59] Markakis E. A. (2002). Development of the neuroendocrine hypothalamus. Front. Neuroendocrinol. 23, 257–291. 10.1016/s0091-3022(02)00003-1 12127306 PMC3242412

[B60] Mathieu J. Barth A. Rosa F. M. Wilson S. W. Peyriéras N. (2002). Distinct and cooperative roles for Nodal and Hedgehog signals during hypothalamic development. Development 129, 3055–3065. 12070082 10.1242/dev.129.13.3055

[B61] Matsuda K. Azuma M. Maruyama K. Shioda S. (2013). Neuroendocrine control of feeding behavior and psychomotor activity by pituitary adenylate cyclase-activating polypeptide (PACAP) in vertebrates. Obes. Res. Clin. Pract. 7, e1–e7. 10.1016/j.orcp.2012.10.002 24331677

[B62] McClellan K. M. Parker K. L. Tobet S. (2006). Development of the ventromedial nucleus of the hypothalamus. Front. Neuroendocrinol. 27, 193–209. 10.1016/j.yfrne.2006.02.002 16603233

[B63] Michaud J. L. (2001). The developmental program of the hypothalamus and its disorders. Clin. Genet. 60, 255–263. 10.1034/j.1399-0004.2001.600402.x 11683768

[B64] Michaud J. L. Boucher F. Melnyk A. Gauthier F. Goshu E. Lévy E.. (2001). Sim1 haploinsufficiency causes hyperphagia, obesity and reduction of the paraventricular nucleus of the hypothalamus. Hum. Mol. Genet. 10, 1465–1473. 10.1093/hmg/10.14.1465 11448938

[B65] Michaud J. L. DeRossi C. May N. R. Holdener B. C. Fan C. M. (2000). ARNT2 acts as the dimerization partner of SIM1 for the development of the hypothalamus. Mech. Dev. 90, 253–261. 10.1016/s0925-4773(99)00328-7 10640708

[B66] Michaud J. L. Rosenquist T. May N. R. Fan C. M. (1998). Development of neuroendocrine lineages requires the bHLH-PAS transcription factor SIM1. Genes Dev. 12, 3264–3275. 10.1101/gad.12.20.3264 9784500 PMC317216

[B67] Moffett P. Pelletier J. (2000). Different transcriptional properties of mSim-1 and mSim-2. FEBS Lett. 466, 80–86. 10.1016/s0014-5793(99)01750-0 10648817

[B68] Münzberg H. Morrison C. D. (2015). Structure, production and signaling of leptin. Metabolism 64, 13–23. 10.1016/j.metabol.2014.09.010 25305050 PMC4267896

[B69] Ogawa S. Ramadasan P. N. Goschorska M. Anantharajah A. Ng K. W. Parhar I. S. (2012). Cloning and expression of tachykinins and their association with kisspeptins in the brains of zebrafish. J. Comp. Neurol. 520, 2991–3012. 10.1002/cne.23103 22430310

[B70] Ohyama K. Ellis P. Kimura S. Placzek M. (2005). Directed differentiation of neural cells to hypothalamic dopaminergic neurons. Development 132, 5185–5197. 10.1242/dev.02094 16284116

[B71] Omura T. Morohashi K. (1995). Gene regulation of steroidogenesis. J. Steroid Biochem. Mol. Biol. 53, 19–25. 10.1016/0960-0760(95)00036-y 7626452

[B72] Parker K. L. Rice D. A. Lala D. S. Ikeda Y. Luo X. Wong M.. (2002). Steroidogenic factor 1: an essential mediator of endocrine development. Recent Prog. Horm. Res. 57, 19–36. 10.1210/rp.57.1.19 12017543

[B73] Parker K. L. Schimmer B. P. (1995). Transcriptional regulation of the genes encoding the cytochrome P-450 steroid hydroxylases. Vitam. Horm. 51, 339–370. 10.1016/s0083-6729(08)61044-4 7483327

[B74] Pearson C. A. Placzek M. (2013). Development of the medial hypothalamus: forming a functional hypothalamic-neurohypophyseal interface. Curr. Top. Dev. Biol. 106, 49–88. 10.1016/B978-0-12-416021-7.00002-X 24290347

[B75] Peng C. Y. Mukhopadhyay A. Jarrett J. C. Yoshikawa K. Kessler J. A. (2012). BMP receptor 1A regulates development of hypothalamic circuits critical for feeding behavior. J. Neurosci. 32, 17211–17224. 10.1523/JNEUROSCI.2484-12.2012 23197713 PMC3589760

[B76] Puelles L. Rubenstein J. L. (1993). Expression patterns of homeobox and other putative regulatory genes in the embryonic mouse forebrain suggest a neuromeric organization. Trends Neurosci. 16, 472–479. 10.1016/0166-2236(93)90080-6 7507621

[B77] Puelles L. Rubenstein J. L. (2003). Forebrain gene expression domains and the evolving prosomeric model. Trends Neurosci. 26, 469–476. 10.1016/s0166-2236(03)00234-0 12948657

[B78] Ramachandrappa S. Raimondo A. Cali A. M. Keogh J. M. Henning E. Saeed S.. (2013). Rare variants in single-minded 1 (SIM1) are associated with severe obesity. J. Clin. Invest. 123, 3042–3050. 10.1172/JCI68016 23778139 PMC3696558

[B79] Ramaswamy S. Seminara S. B. Ali B. Ciofi P. Amin N. A. Plant T. M. (2010). Neurokinin B stimulates GnRH release in the male monkey (Macaca mulatta) and is colocalized with kisspeptin in the arcuate nucleus. Endocrinology 151, 4494–4503. 10.1210/en.2010-0223 20573725 PMC2940495

[B80] Russek-Blum N. Gutnick A. Nabel-Rosen H. Blechman J. Staudt N. Dorsky R. I.. (2008). Dopaminergic neuronal cluster size is determined during early forebrain patterning. Development 135, 3401–3413. 10.1242/dev.024232 18799544 PMC2692842

[B81] Ryu S. Mahler J. Acampora D. Holzschuh J. Erhardt S. Omodei D.. (2007). Orthopedia homeodomain protein is essential for diencephalic dopaminergic neuron development. Curr. Biol. 17, 873–880. 10.1016/j.cub.2007.04.003 17481897

[B82] Schonemann M. D. Ryan A. K. McEvilly R. J. O’Connell S. M. Arias C. A. Kalla K. A.. (1995). Development and survival of the endocrine hypothalamus and posterior pituitary gland requires the neuronal POU domain factor Brn-2. Genes Dev. 9, 3122–3135. 10.1101/gad.9.24.3122 8543156

[B83] Sekido R. Lovell-Badge R. (2008). Sex determination involves synergistic action of SRY and SF1 on a specific Sox9 enhancer. Nature 453, 930–934. 10.1038/nature06944 18454134

[B85] Servili A. Le Page Y. Leprince J. Caraty A. Escobar S. Parhar I. S.. (2011). Organization of two independent kisspeptin systems derived from evolutionary-ancient kiss genes in the brain of zebrafish. Endocrinology 152, 1527–1540. 10.1210/en.2010-0948 21325050

[B86] Shinoda K. Lei H. Yoshii H. Nomura M. Nagano M. Shiba H.. (1995). Developmental defects of the ventromedial hypothalamic nucleus and pituitary gonadotroph in the Ftz-F1 disrupted mice. Dev. Dyn. 204, 22–29. 10.1002/aja.1002040104 8563022

[B87] Silveira L. F. Trarbach E. B. Latronico A. C. (2010). Genetics basis for GnRH-dependent pubertal disorders in humans. Mol. Cell. Endocrinol. 324, 30–38. 10.1016/j.mce.2010.02.023 20188792

[B88] Simeone A. D’Apice M. R. Nigro V. Casanova J. Graziani F. Acampora D.. (1994). Orthopedia, a novel homeobox-containing gene expressed in the developing CNS of both mouse and *Drosophila*. Neuron 13, 83–101. 10.1016/0896-6273(94)90461-8 7913821

[B89] Suárez R. Gobius I. Richards L. J. (2014). Evolution and development of interhemispheric connections in the vertebrate forebrain. Front. Hum. Neurosci. 8:497. 10.3389/fnhum.2014.00497 25071525 PMC4094842

[B90] Swaab D. F. (2004). Neuropeptides in hypothalamic neuronal disorders. Int. Rev. Cytol. 240, 305–375. 10.1016/s0074-7696(04)40003-5 15548416

[B91] Szabó N. E. Zhao T. Cankaya M. Theil T. Zhou X. Alvarez-Bolado G. (2009). Role of neuroepithelial Sonic hedgehog in hypothalamic patterning. J. Neurosci. 29, 6989–7002. 10.1523/JNEUROSCI.1089-09.2009 19474326 PMC6665591

[B92] Szarek E. Cheah P. S. Schwartz J. Thomas P. (2010). Molecular genetics of the developing neuroendocrine hypothalamus. Mol. Cell. Endocrinol. 323, 115–123. 10.1016/j.mce.2010.04.002 20385202

[B93] Takase M. Nakajima T. Nakamura M. (2000). FTZ-F1α is expressed in the developing gonad of frogs. Biochim. Biophys. Acta 1494, 195–200. 10.1016/s0167-4781(00)00201-3 11072086

[B94] Takayanagi Y. Onaka T. (2010). Roles of prolactin-releasing peptide and RFamide related peptides in the control of stress and food intake. FEBS J. 277, 4998–5005. 10.1111/j.1742-4658.2010.07932.x 21126313

[B95] Tessmar-Raible K. Raible F. Christodoulou F. Guy K. Rembold M. Hausen H.. (2007). Conserved sensory-neurosecretory cell types in annelid and fish forebrain: insights into hypothalamus evolution. Cell 129, 1389–1400. 10.1016/j.cell.2007.04.041 17604726

[B96] Tolson K. P. Gemelli T. Gautron L. Elmquist J. K. Zinn A. R. Kublaoui B. M. (2010). Postnatal Sim1 deficiency causes hyperphagic obesity and reduced Mc4r and oxytocin expression. J. Neurosci. 30, 3803–3812. 10.1523/JNEUROSCI.5444-09.2010 20220015 PMC3285557

[B97] Tran P. V. Akana S. F. Malkovska I. Dallman M. F. Parada L. F. Ingraham H. A. (2006). Diminished hypothalamic bdnf expression and impaired VMH function are associated with reduced SF-1 gene dosage. J. Comp. Neurol. 498, 637–648. 10.1002/cne.21070 16917842

[B98] Tran P. V. Lee M. B. Marin O. Xu B. Jones K. R. Reichardt L. F.. (2003). Requirement of the orphan nuclear receptor SF-1 in terminal differentiation of ventromedial hypothalamic neurons. Mol. Cell. Neurosci. 22, 441–453. 10.1016/s1044-7431(03)00027-7 12727442 PMC2710097

[B99] Twan W. H. Hwang J. S. Lee Y. H. Jeng S. R. Yueh W. S. Tung Y. H.. (2006). The presence and ancestral role of gonadotropin-releasing hormone in the reproduction of scleractinian coral, Euphyllia ancora. Endocrinology 147, 397–406. 10.1210/en.2005-0584 16195400

[B100] Umesono Y. Watanabe K. Agata K. (1997). A planarian orthopedia homolog is specifically expressed in the branch region of both the mature and regenerating brain. Dev. Growth Differ. 39, 723–727. 10.1046/j.1440-169x.1997.t01-5-00008.x 9493832

[B101] Unger J. L. Glasgow E. (2003). Expression of isotocin-neurophysin mRNA in developing zebrafish. Gene Expr. Patterns 3, 105–108. 10.1016/s1567-133x(02)00064-9 12609611

[B102] Vandepoele K. De Vos W. Taylor J. S. Meyer A. Van de Peer Y. (2004). Major events in the genome evolution of vertebrates: paranome age and size differ considerably between ray-finned fishes and land vertebrates. Proc. Natl. Acad. Sci. U S A 101, 1638–1643. 10.1073/pnas.0307968100 14757817 PMC341801

[B103] Wang W. Lufkin T. (2000). The murine Otp homeobox gene plays an essential role in the specification of neuronal cell lineages in the developing hypothalamus. Dev. Biol. 227, 432–449. 10.1006/dbio.2000.9902 11071765

[B104] Wircer E. Ben-Dor S. Levkowitz G. (in press). “Non mammalian models for neurohypophyseal peptides,” in International Neuroendocrine Federation (INF) Masterclass Series: Molecular Neuroendocrinology: “From Genome to Physiology”, eds Murphy D. Gainer H. (Chichester: Wiley & Sons, Ltd).

[B105] Wolf A. Ryu S. (2013). Specification of posterior hypothalamic neurons requires coordinated activities of Fezf2, Otp, Sim1a and Foxb1.2. Development 140, 1762–1773. 10.1242/dev.085357 23533176

[B106] Woods S. Farrall A. Procko C. Whitelaw M. L. (2008). The bHLH/Per-Arnt-Sim transcription factor SIM2 regulates muscle transcript myomesin2 via a novel, non-canonical E-box sequence. Nucleic Acids Res. 36, 3716–3727. 10.1093/nar/gkn247 18480125 PMC2441813

[B107] Wright D. A. Li T. Yang B. Spalding M. H. (2014). TALEN-mediated genome editing: prospects and perspectives. Biochem. J. 462, 15–24. 10.1042/BJ20140295 25057889

[B114] Wullimann M. F. Rink E. (2001). Detailed immunohistology of Pax6 protein and tyrosine hydroxylase in the early zebrafish brain suggests role of Pax6 gene in development of dopaminergic diencephalic neurons. Brain Res. Dev. Brain Res. 131, 173–191. 11718849 10.1016/s0165-3806(01)00270-x

[B115] Wullimann M. F. Rink E. (2002). The teleostean forebrain: a comparative and developmental view based on early proliferation, Pax6 activity and catecholaminergic organization. Brain Res. Bull. 57, 363–370. 11922990 10.1016/s0361-9230(01)00666-9

[B108] Xi D. Gandhi N. Lai M. Kublaoui B. M. (2012). Ablation of Sim1 neurons causes obesity through hyperphagia and reduced energy expenditure. PLoS One 7:e36453. 10.1371/journal.pone.0036453 22558467 PMC3338647

[B109] Xi D. Roizen J. Lai M. Gandhi N. Kublaoui B. (2013). Paraventricular nucleus Sim1 neuron ablation mediated obesity is resistant to high fat diet. PLoS One 8:e81087. 10.1371/journal.pone.0081087 24260538 PMC3834298

[B110] Xu B. Goulding E. H. Zang K. Cepoi D. Cone R. D. Jones K. R.. (2003). Brain-derived neurotrophic factor regulates energy balance downstream of melanocortin-4 receptor. Nat. Neurosci. 6, 736–742. 10.1038/nn1073 12796784 PMC2710100

[B111] Yang N. Dong Z. Guo S. (2012). Fezf2 regulates multilineage neuronal differentiation through activating basic helix-loop-helix and homeodomain genes in the zebrafish ventral forebrain. J. Neurosci. 32, 10940–10948. 10.1523/JNEUROSCI.2216-12.2012 22875928 PMC3478895

[B112] Zhao L. Kim K. W. Ikeda Y. Anderson K. K. Beck L. Chase S.. (2008). Central nervous system-specific knockout of steroidogenic factor 1 results in increased anxiety-like behavior. Mol. Endocrinol. 22, 1403–1415. 10.1210/me.2008-0034 18372344 PMC2422821

